# On the Presence of Uric Acid in the Kidneys, as a Sign of a Child Having Been Born Alive

**Published:** 1849-12

**Authors:** 


*On the Presence of Uric Acid in the Kidneys, as a Sign of a Child having been born alive.* By Dr. Virchow.—Dr. Virchow, in the Transactions of the Berlin Obstetrical Society, has proposed a sign, to ascertain whether a child has been born alive; or, rather, whether it lived more than two days. The test is to be found in the absence or presence of uric acid in the kidneys.

Dr. Virchow wishes to show that the presence of uric acid in the kidney (at once to be detected by the naked eye), is a conclusive proof that the child found dead has been born alive. The ancient anatomists remarked a red or yellowish substance coating the mammilla of the kidneys of infants, and which modern science has found to be uric acid, and analogous to the calculi sometimes found in the bladders of children. Schlosseberger treats of it under the name of "Infractum acidi urici;" and it is easily discovered by making a transverse section of the kidney, when a considerable number of yellowish-brown, and sometimes light yellow, rays of this substance, are seen ramifying from the tubular, and sometimes from the cortical substance to the mammilla. Under the microscope, we make out sometimes solid cylindrical particles, of a yellowish-brown color, though more frequently these cylinders seem to be not yet formed, or to be broken up into molecules of a darker color, round or angular, and not unlike the crystals of urate of ammonia. A greater quantity is obtained by pressure. The conclusions of Dr. Virchow are:

1. That the deposit is never found in children who have been born dead, or who have died within forty-eight hours after birth.
2. That the deposit is not found, or does not occur, until about forty-eight hours after birth.
3. That the deposit is not generally found later than the twentieth day after birth.

There are, however, exceptions to these rules; for the deposit was, in one case, found on the twenty-ninth day after birth; and Dr. Virchow had not found it in an infant dead on the tenth day; nor in another on the thirteenth; nor in a third on the sixth day; these, however, may be the consequence of disease. The subject is now left in the hands of British observers, who, I trust, will soon determine the value of Dr. Virchow's views.—[Abridged from Dr. Bushman, in Medical Times for January 20, 1849.]—*Ib.*

